# Cases at the Louisville Marine Hospital

**Published:** 1850-03

**Authors:** David W. Yandell

**Affiliations:** Louisville, Ky., Attending Physician for the Faculty at the Hospital


Art. III.—*Cases at the Louisville Marine Hospital.* By David W. Yandell, M. D., of Louisville, Ky., Attending Physician for the Faculty at the Hospital.

*Case* 1. *Gastritis—death after seventeen days illness—gastric mucous membrane red and friable.* Michael Brosman, æt. 30, laborer, a hard drinker, was admitted into the hospital on the 29th of January. Ten days before, after having been on a long "spree," he was seized with severe diarrhea. This was in New Orleans. The next day he took a boat for Louisville. Notwithstanding the free use that he made of brandy and water to check the disease, instead of improving, he grew worse, and on the fifth day from port he felt an acute pain in the epigastrium, and soon after everything he swallowed was rejected. His discharges grew less and less frequent, and at the end of forty-eight hours, had entirely ceased. He vomited almost incessantly small quantities of bilious matter, which he described as being excessively bitter. All drinks which he endeavored to take, were instantly rejected. He had very acute pain over the epigastrium, while the remainder of abdomen was entirely free from it. His thirst, which was almost intolerable, he feared to satisfy, because the smallest quantity of water taken into the stomach produced vomiting. He supposed he must have had fever, though he never counted his pulse. His skin was very dry and hot—his mouth parched and burning. During the trip from New Orleans, three deck passengers died from cholera. He had had slight disposition to general cramp, and actually suffered several times with very painful cramps of his hands and feet. He was unable to sleep; had slept none for several days in succession; had been troubled with frightful sights, one of which, in particular, had greatly distressed and alarmed him,—a horse without a head, that appeared to be standing on the foot of his bed. Five days passed on in this way; the boat reached Louisville, and the patient was brought to the hospital. The following was his state at this time:—

His countenance was pale, and expression both anxious and heavy; he was very feeble, and spoke with a faint husky voice. The tongue was covered with a white coat—intensely red at edges and tip, dry and cool. No stools for three days. Patient had vomited repeatedly in the preceding twenty-four hours; all ingesta were promptly ejected. Thirst; anorexia; cephalalgia; mouth dry and parched; skin relaxed, cool and covered with a clammy perspiration. Pulse was 90, small and very feeble. Respiration laborious, heavy and slightly accelerated. Intellectual faculties obtuse. Hands and feet corrugated. Patient complained of great pain over the region of the stomach, where the tenderness was exquisite on pressure. The remaining abdominal viscera appeared, on palpation at least, healthy.

My prescriptions for the morning were small quantities of calomel and morphine, given at short intervals until sleep was procured; leeches to the epigastrium, to be followed, if it did not, by its weight, excite too much pain, by a flaxseed poultice; in the event of its being disagreeable, cloths, wrung out of warm water, were to be applied, in order to assist the bleeding from the leech-bites. Small pellets of ice were allowed the patient as often as he desired, and this was the case almost incessantly. At bedtime, if the vomiting continued and the patient had slept none, the quantity of morphine was to be increased and mustard sinapisms were to be applied to the spine over the origin of the gastric nerves. A stimulating cathartic injection to be administered immediately.

On the following day there was a slight amendment; the vomiting had not altogether ceased, but it was less copious and less frequent; the patient had been able to retain a small quantity of mucilage that he had taken; the pain in the stomach was less; the tenderness very slightly, if at all, diminished. Bowels moved twice; stools thin and yellowish. The patient slept a few hours —awoke delirious, and continued somewhat so at visit. Pulse 100; skin not so cool; dry. Other symptoms materially the same as on the day before. ℞. Leeches, anodyne injections, ice, cloths wrung out of ice-water and ice in substance over the stomach. Omit calomel, and give morphine only, every few hours.

During the next twenty-four hours, the state of the patient, in some respects, steadily improved. The vomiting ceased; the pain and tenderness disappeared; the thirst abated; the delirium passed away. Still the pulse retained its frequency and adynamic characters; the tongue continued red, and had become dry and coated with a brownish fur; the teeth were covered with sordes; the respiration was not less laborious; the countenance remained unchanged, and the patient expressed himself as feeling no better. His bowels had been operated on but once. Continue ice; omit other prescriptions.

The condition of the patient now assumed the characters so universally observed, where cholera prevailed, which followed violent attacks of that disease The generally low, muttering delirium, alternating with extreme sluggishness of the intellectual faculties; the hot, dry skin; the quick, small and feeble pulse; the dry and parched mouth; the glazed and dry tongue; the intense thirst; the complete anorexia, and the extreme physical weakness which so constantly supervene in severe cases of cholera, after the acute symptoms have subsided, and especially when the disease is passing to a fatal termination, were all present in this instance.

All appreciable physical, and, indeed, rational signs of gastritis disappeared for the space of four days, during which time the symptoms were those above described, when, on the morning of the 8th of February, the patient complained of pain in the pit of the stomach, and this organ again became tender on pressure.

On the 7th, copious epistaxis set in, and continued, notwithstanding the repeated application of cold and astringents, and the internal administration of these latter substances in large quantities, for thirty-six hours; when the house pupil deemed it necessary to resort to tamponing the posterior nares, by which the hemorrhage was arrested. During this time the nurse thinks that the patient must have lost two quarts of blood.

At the clinic, on the afternoon of Friday, (the 8th) the condition of the patient was as follows: Great pain and tenderness, on pressure, in epigastrium; skin cool; pulse 115, small and thready; face heavy; mind dull; thirst; loathing of food; voice feeble; respiration labored; mouth and lips dry; inside of mouth and fauces painful, and covered by a distinct diptheritic eruption; tongue dry, glazed, brownish in centre, and intensely red at edges and tip. Three alvine dejections in preceding twenty-four hours; thin and watery. Great increase of debility seemed to accompany the development of the eruption.

Prof. Bartlett coincided with me in the use of a large blister over the stomach, and the administration, at intervals of four hours, of six grains of calomel and half a grain of morphine.

That night the features became perceptibly altered; the pulse continued frequent; the voice grew more and more feeble, hiccough, vomiting from time to time, wandering delirium, and the patient rapidly sank till Sunday, the 10th, when, at 11 o'clock, he died.

*Postmortem Examination.*—Mucous membrane of the stomach of a bright red color, along lesser curvature and pyloric orifice, and of a dark grayish hue over remaining portion; softened throughout. Mucous membrane of duodenum, at its superior third, red and somewhat softened.

In this case, the epigastrium was the seat of an acute pain, which did not, however, as in most instances, continue throughout the entire course of the disease, there being an interval of four or five days, during which the inflammation had evidently been partially subdued, when there was no pain whatever complained of by the patient.

There is a case related by Andral, in his Clinic, of gastritis arising from over-eating during convalescence from cholera, in which the following train of symptoms was observed, without there being the *slightest pain in the epigastrium:* eyes dull and sunk, as when he had cholera; tongue presented nothing particular; burning thirst; vomiting; no pain in epigastrium or rest of abdomen; pulse frequent; skin not hot; some liquid stools.

For twenty-five days, patient remained in a state of continued fever, with tongue constantly dry and red; burning thirst; constant nausea; occasionally vomited either thready mucus, or yellow or greenish bile. *Abdomen, including epigastrium, was free from pain in all its points.* Obstinate constipation. Died, as if exhausted, without a struggle and with intellect perfect.

It was my misfortune, last summer, during the prevalence of the cholera, to meet with cases corresponding to the one which I have condensed from the great work of Andral; but, at the time, being unable to make a note of them, I cannot report them.

Touching the symptoms of gastritis, Andral holds the following language: "There is no symptom necessarily connected with the disease which it indicates in very many cases, and there may be acute gastritis without vomiting and without pain, as there are instances of pleuritis without stitch in the side, and pneumonia, without the rust-colored expectoration."

*Case 2. Wound of the Brain—Epileptiform symptoms—Death three months after—Abscesses in the Brain.*—Matthias Gross, æt. 27, born in Strasbourg, of feeble health and dissipated habits, was received into hospital on Nov. 27th, for traumatic inflammation of left eyelid. Having had, on the evening of admission, several slight, short, epileptiform attacks, he was transferred to medical wards, where I first saw him on the morning of the next day.—His physiognomy was stupid, almost fatuous; mind same; answered first questions in French; soon after, run off into a low, listless tone, in German. Surface of body pale and cool. Pulse 60, feeble. Tongue furred on middle and tip, edges clean and smooth; moist. Says his appetite is good; no thirst; bowels costive. Complains of dull heavy pain in the head—constant, but more severe at one time than at another. Gets up in the night and wanders about; does not seem to know what he wants. Has fits at short intervals during the day—they were also brought on by lifting him up in bed, and by addressing him suddenly in a sharp tone of voice. They were slight and lasted but a few minutes.

The wound of the eyelid excited no attention, until one day the patient told me that three months before his admission into the hospital, it had been inflicted with a pocket knife in a street brawl. He had hardly stated this much, when he was seized with a convulsion, and all further replies to my questions became impossible. A careful examination of the wound only revealed that in depth it reached to the bone, and its width was equal to that of the large blade of a common Congress pocket-knife. At the visit, on the following day, I learned that the patient had received, on the same occasion, a stab in the abdomen, which confined him to his bed for two weeks. A few days before leaving his bed, he had several convulsions. They were slight, however, and only of a moment's duration—so slight and so brief that they did not excite his attention, until he found that they gradually increased in frequency; and, finally, during the four or five succeeding weeks, at least, they amounted to fifteen or

[illegible]nty during the twenty-four hours. The general health became impaired. He had no appetite; could get no sleep; emaciated rapidly, and lost his mind.

The general appearance and air of the patient led Dr. Bartlett to suspect that he might be guilty of onanism.—He consequently desired me to have him carefully watched. Although this was done, for some time, no proof was discovered of indulgence in this practice.

Things wore gradually on. The patient lost all power of intelligible speech, and mumbled answers to my questions, each attempt to speak being followed immediately by a convulsion. The symptoms grew daily worse; hectic fever and night sweats came on, and soon after colliquative diarrhea; the pulse became more and more feeble; the intellectual faculties were finally altogether destroyed, and the patient, after lying for two days in a state of profound stupor, died on the

*Postmortem Examination.*—On removing the skull cap, a knife-blade, an inch and a quarter in length was found projecting into the substance of the brain. It had entered the cavity of the cranium on a line with the superior and inner third of the orbital plate of the frontal bone just below the superciliary ridge, and was so firmly imbedded in the bone that it was impossible to remove it.—Externally the blade had been broken off on a line with the bone, which accounts for its not having been discovered in the examination of the wound. Three large abscesses, communicating with each other, were found in the left hemisphere of the cerebrum—the first situated at the point of the blade, the other two placed below and internal to it. The greater part of the cerebral mass upon that side, was in a softened and even pulpy condition.—The other organs healthy.

Louisville, Ky., Feb. 20, 1850.

