# Erratum

**Published:** 1850-04

**Authors:** 


*Erratum.*—In my communication for the March number of the Journal I find that the date of the death of Mathias Gross is omitted. He died on the 18th December.

